# Supplementary material for: Complexome profiling of the Chlamydomonas psb28 mutant reveals TEF5 as an early PSII assembly factor
Source: Plant Cell. 2025 Mar 17;37(6):koaf055. doi: 10.1093/plcell/koaf055 (PMC12164590; doi:10.1093/plcell/koaf055)
Supplement: koaf055_Supplementary_Data [file koaf055_supplementary_data.zip › psb28_interactive.zip!/plots/intensity/qualityPlots/peptides/_Cre17.g732250.t1.1.html]

### Cre17.g732250.t1.1

Trivial name:   
SeqCoverage [median]: 35.40  
Mass: 16.48 kDa  
  
 

| Sequence | MissedCleavage | Mass [Da] | wtIntensity | muIntensity | Score |
| --- | --- | --- | --- | --- | --- |
| EFTSHGAR | 0 | 903.42 | 0.00 | 1984.77 | 111.82 |
| IIESDAR | 0 | 802.42 | 0.00 | 11731.73 | 134.66 |
| LVGPEDQPDLFEGENMETLGK | 0 | 2317.08 | 13912.99 | 30927.82 | 141.83 |
| QGEASTITPPPSNEKPTVVR | 1 | 2107.09 | 11660.58 | 21735.29 | 137.05 |
|  |  |  | 25573.6 | 66379.6 |  |
